# Thermogenic Fat: Development, Physiological Function, and Therapeutic Potential

**DOI:** 10.3390/ijms22115906

**Published:** 2021-05-31

**Authors:** Bruna B. Brandão, Ankita Poojari, Atefeh Rabiee

**Affiliations:** 1 Section of Integrative Physiology and Metabolism, Joslin Diabetes Center, Harvard Medical School, Boston, MA 02215, USA; bruna.brasilbrandao@joslin.harvard.edu; 2 Department of Physiology & Pharmacology, Thomas J. Long School of Pharmacy & Health Sciences, University of the Pacific, Stockton, CA 95211, USA; a_poojari@u.pacific.edu

**Keywords:** adipose tissue, development, molecular circuits, secretome, thermogenesis, metabolism, obesity, therapy

## Abstract

The concerning worldwide increase of obesity and chronic metabolic diseases, such as T2D, dyslipidemia, and cardiovascular disease, motivates further investigations into preventive and alternative therapeutic approaches. Over the past decade, there has been growing evidence that the formation and activation of thermogenic adipocytes (brown and beige) may serve as therapy to treat obesity and its associated diseases owing to its capacity to increase energy expenditure and to modulate circulating lipids and glucose levels. Thus, understanding the molecular mechanism of brown and beige adipocytes formation and activation will facilitate the development of strategies to combat metabolic disorders. Here, we provide a comprehensive overview of pathways and players involved in the development of brown and beige fat, as well as the role of thermogenic adipocytes in energy homeostasis and metabolism. Furthermore, we discuss the alterations in brown and beige adipose tissue function during obesity and explore the therapeutic potential of thermogenic activation to treat metabolic syndrome.

## 1. Introduction

Obesity is the main driver of insulin resistance (IR), type two diabetes (T2D), and metabolic syndrome. Obese subjects, especially the ones with a high percentage of intra-abdominal fat, have a greater risk of developing cardiovascular disease (CVD), the leading cause of death in industrial countries [1]. The prevalence of obesity is multifactorial and includes socioeconomic, educational status, issues concerning mental health, genetics, sedentarism, and diet [2]. It is now appreciated that obesity develops when energy consumption (food intake) overcomes energy expenditure. This induces white adipose tissue (WAT) expansion followed by reduced mass and activity of brown/beige adipocytes (fat cells), thereby contributing to the development of metabolic disorders during obesity [3].

WAT is the principal site for energy storage, while brown and beige adipocytes are the sites for energy expenditure (EE) due to their thermogenic capacity [4]. Adipose tissue (AT) is also an important endocrine organ responsible for the secretion of many molecules, including lipids [5,6], proteins [7,8,9], and miRNAs [10]. These factors serve as paracrine-endocrine signals, critical for the function of AT itself, as well as non-adipose tissues, regulation of whole-body metabolism, and insulin sensitivity [11]. Therefore, interventions that can induce the formation and activation of brown and beige adipocytes such as cold exposure [12], pharmacological activation of the adrenergic pathways [13], or even genetic manipulation of adipocytes [14] are attractive therapies to improve metabolic health in obese humans.

## 2. Adipose Tissue Development and Origin

Adipocytes are categorized as white or brown depending on their function and morphological characteristics. As an endocrine tissue responsible for hormonal secretion, WAT plays a role in fatty acid (FA) biosynthesis by storing triglyceride (TG), and it is composed of unilocular adipocytes containing a large lipid droplet. By comparison, brown adipose tissue (BAT) plays a role in glucose uptake and FA breakdown, leading to energy dissipation and heat production. Brown adipocytes are multilocular cells with central nuclei and mitochondria rich in the expression of uncoupling protein 1 (Ucp1) [15,16,17]. The primary function of BAT is non-shivering thermogenesis, an energy-intensive process in which chemical fuel is turned into physical heat. In addition to BAT, the process of thermogenesis can also be carried out by a third type of adipocytes known as beige adipocytes. Beige adipocytes share many of their morphological features with brown adipocytes namely the presence of multilocular lipid droplets and abundant mitochondria expressing Ucp1 [15,18]. However, unlike brown adipocytes, which are committed to the process of thermogenesis, beige adipocytes are a form of thermogenic adipocytes that may be induced within WAT depots sporadically via a white-to-brown transition known as “browning”. The extreme plasticity of beige adipocytes causes the browning process to be reversible and highly dependent on the continuation of energy imbalance caused by external cues. Interestingly, the induced beige adipocytes transition to their original white state soon after the energy balance is restored [19,20,21,22,23,24]. 

Even though WAT itself may be categorized broadly into two subtypes of visceral white adipose tissue (vWAT) and subcutaneous white adipose tissue (scWAT), beige adipocytes are mainly known to be induced within scWAT depots [16]. vWAT comprises perirenal (prvWAT), perigonadal (pgvWAT), mesenteric (mvWAT), and retroperitoneal (rpvWAT) white adipose tissues. In humans, there are scWAT depots in the cranial, nasal, gastrointestinal, femoral, and gluteal areas. Correspondingly, such scWAT depots are also found in rodents in the anterior subcutaneous white adipose tissues (ascWAT) and the posterior subcutaneous (pscWAT) which include inguinal, dorso-lumbal, and gluteal WAT [15,25]. Anatomically, BAT depots are dispersed in the scapulae (interscapular, cervical, and axillary) and thoracic (mediastinal) areas of mice and rats [26]. As opposed to the previously held view, that BAT is only present in the neck and shoulder regions of newborn children and infants [27], it has been widely proven by various studies, that active BAT depots with thermogenic capacity are found lying between anterior neck muscles and in the paracervical and supraclavicular regions of adult humans [27,28,29,30,31]. 

The timeline for the formation of BAT and WAT during embryogenesis also varies with the emergence and development of BAT occurring earlier in all mammalian species compared to WAT. In humans, BAT formation starts at the second gestational trimester where it is observed mainly in the head and neck and it later develops in the trunk, upper, and lower limb regions as well. In rodents, the formation of interscapular BAT occurs between E15–16 (embryonic phase) and increases postnatally between P15–21. Functional BAT formation with the ability to carry out thermogenesis is completed 2 days before birth during the E18–19 [32,33,34,35]. WAT development also occurs prenatally and the formation of both scWAT and vWAT is completed at the end of gestational weeks 23 and 28, respectively [36]. Unlike humans, wherein the formation of WAT is initiated and mostly completed in utero, for rodents, the development of scWAT and vWAT is mostly postnatal. vWAT formation commences after birth and scWAT formation is completed 56 days postpartum [37,38,39].

Brown and white AT originate and evolve from the mesoderm. vWAT arises from intermediate and lateral plate mesoderm [40,41,42,43], while BAT originates from paraxial mesoderm [44]. The origin of scWAT is still under dispute, with evidence indicating that the progenitor cells for this AT originate from both mesoderm and neuroectoderm [45,46,47,48]. Additionally, each type of fat depot consists of distinct and different precursor/progenitor populations that are regulated by various factors affected by age, gender, and environmental conditions. The recent advances in lineage tracing strategies, as well as gene expression studies, showed that white and brown adipocytes originate from different mesenchymal stem cells (MSCs). In brief, vWAT depots are largely derived from progenitors expressing Wilms tumor 1 (Wt1) [42,49,50] and scWAT depots mainly originate from paired related homeobox 1 (Prx1) expressing progenitors [51,52,53,54,55]. Although in the past, it was believed that myogenic factor 5 (Myf5), paired box 3 (Pax3), and paired box 7 (Pax7) expressing progenitors were only responsible for the formation of BAT, it is now understood that scWAT depots of the dorsal–anterior body regions also partly share their origin with the above-listed progenitors [17,47,56,57,58,59]. Thus, it may be noted that even though adipocytes may develop from a common lineage, they may or may not have similar functions. 

Several studies have noted that the existence of various cell surface markers may be used as a strategy to isolate beige and brown progenitors [60]. In humans, it was found that Cd34+/Cd31- and Cd34+/Cd146-/Cd45-/Cd56- cells were BAT progenitors in small vessels and fetal muscles, respectively [61]. Cd29+/Cd31-/Cd34-/Cd45- progenitor cells lead differentiation to the beige adipocytes within the scWAT depots of humans [62]. Moreover, beige adipocytes progenitors in mouse scWAT are marked by Cd81+/Sca1/Lin- and the Cd81+/Pdgfα+/Lin- mark the beige progenitors in human scWAT [63]. 

One can assume from these studies that Cd34 and Cd81 may be used as markers to identify brown and beige progenitors, respectively. However, isolation of different adipocytes based on such cell surface markers must be done cautiously considering factors such as the location of the depots within which the progenitors have resided and the effect of the surrounding microenvironment [64]. It is also known that adipocyte progenitors express platelet-derived growth factor receptors α (Pdgfrα) and β (Pdgfrβ) [65]. In ex vivo studies, the presence of both Pdgfrα and Pdgfrβ in adipose stromal cells (ASCs) was confirmed [66]. However, in adult mice, the progenitors only expressed either Pdgfrα or Pdgfrβ [67,68]. Using Pdgfrα Cre recombinase mouse models, the existence of the Pdgfrα expressing cells during the normal establishment of WAT was shown. However, the existence of multiple Pdgfrα+ populations that some of them are not adipogenic further indicates that Pdgfrα expression alone cannot be used to identify adipocyte progenitor populations within WAT [37]. Studies in humans and mice have confirmed that commitment towards either beige or white adipogenesis is predetermined by the balance between Pdgfrα and Pdgfrβ signaling in adipocyte progenitors, and a high level of Pdgfrα expression precedes ASCs differentiation into beige adipocytes. Moreover, in vitro studies showed that during the initial stages of adipocyte lineage development, Pdgfrβ signaling promotes white adipogenesis, whereas Pdgfrα signaling is followed by brown adipogenesis [69]. As a result of impaired β-adrenergic signaling (a common cue for initiation of browning), a subset of Pdgfrα+/Cd34+/Cd29+ progenitors in scWAT expresses myoblast determination protein (MyoD); which supports beige adipogenesis following cold acclimation. However, these MyoD derived beige adipocytes are different from standard beige adipocytes in terms of their developmental origin and their metabolism with these beige adipocytes having enhanced glucose metabolism and therefore, named as glycolytic beige adipocyte. It is now postulated that multiple subtypes of beige thermogenic adipocytes exist and their functions vary based on the nature of external stimuli, such as cold acclimation or diet [70].

## 3. Molecular Circuits Regulating Brown and Beige Adipose Tissue Development and Function

Adipocyte differentiation happens when multipotent stem cells commit to forming preadipocytes that further undergo terminal differentiation to form mature adipocytes. Despite the differences in developmental origins of brown and beige adipocytes, both cell types share a similar transcriptional cascade involving a distinct chromatin landscape governing a vast gene expression program that controls the process of fat differentiation. The chromatin landscape itself comprises an intricate and complex network of transcriptional regulators (transcription factors and cofactors), epigenetic factors (histone marks and chromatin methylation), and non-coding RNAs (long non-coding RNAs and microRNAs).

### 3.1. Transcriptional Regulation of Brown and Beige Adipocytes 

Transcription factors (TFs) are DNA binding proteins that activate or repress RNA polymerase II (Pol II)-mediated transcription. These proteins bind to DNA sequences at promoter or other regulatory regions such as enhancers. The core transcriptional machinery coordinated by peroxisome proliferator-activated receptor gamma (Pparγ) and members of the C/ebp family of transcription factors (TFs) governing the differentiation process of adipocytes are similar for all types of fat cells and have been extensively discussed elsewhere [16,71,72,73]. Various TFs, cofactors (corepressors and coactivators), and nuclear receptors (NRs) which regulate the process of white versus brown lineage commitment are already known and coordinate functionally in a concerted manner to modulate the principal adipogenic transcriptional machinery. Overall, the process of formation of beige and brown AT by TFs is mainly regulated via a two-fold mechanism, i.e., either activation of BAT and beige-selective genes or by suppressing WAT-specific genes. Here, we describe the main brown and beige fat-selective signatures of TFs. 

Early β-cell factor 2 (Ebf2) is a marker of committed brown adipocytes that inhibits the expression of MyoD and muscle-specific transcription factor (myogenin) [33,74]. The high expression level of Ebf2 in adult human brown preadipocytes suggests its role in brown preadipocyte determination [75]. Ebf2 also promotes brown adipocytes differentiation by recruiting Pparγ to its BAT-selective binding sites [74]. Ebf2 knockdown diminished the brown fat-specific features of BAT [74]. Besides, Ebf2 overexpression in WAT induces browning and thermogenesis [76].

Ewing sarcoma (Ews) regulates the expression of bone morphogenic protein 7 (Bmp7) and thereby plays an important role in the commitment of early mesenchymal progenitors to brown adipocytes. Ews is also involved in the differentiation process of BAT as the brown preadipocytes isolated from the newborn Ews null mice did not differentiate ex vivo. In addition, decreased number of multilocular lipid droplets and mitochondria, as well as reduced Ucp1 expression in the BAT of Ews null mice, indicates the critical role of Ews in brown fat phenotype and thermogenic function. Ews also plays role in the browning of WAT as the Ews heterozygous mice showed fewer beige cells formed in the WAT exposed to the browning stimuli such as Pparγ agonists and β3-adrenergic stimulation [77]. The role of Ews in controlling the thermogenic function of beige and brown AT is proposed to be via stabilizing Pgc1α [78]. 

Y box binding protein 1 (Ybx1) is a cold shock domain protein that together with Ews regulates the Bmp7 expression through which plays a role in the commitment of precursor cells to BAT. In the same complex with Ews, Ybx1 also regulates the differentiation of brown preadipocytes [77]. We recently demonstrated a critical role of Ybx1 in priming and maintaining the thermogenic capacity during adipogenesis [79].

Heat shock factor 1 (Hsf1) deficient mice are more sensitive to low temperatures, reduced Ucp1 expression in scWAT and BAT, and decreased thermogenesis and β-oxidation indicating an overall reduced brown and beige tissue functionality [80,81].

TATA-binding protein-associated factor 7L (Taf7l), the study by Zhou et al. performed in mice as well as in cell lines introduced the Taf7l as a commitment factor that enhances the brown fat lineage as compared to muscle. Taf7l mediates the loop formation in chromatin bringing together the distal enhancer regions and the promoters, and in that way controls the expression of BAT-selective genes [82].

Zinc finger in the cerebellum 1 (Zic1) has been described with a controversial role in beige and BAT formation. Overexpression of Zic1 in C3H10T1/2 mouse MSCs attenuated the expression of BAT-selective genes and increased the expression of myogenic genes [83]. In mice, however, the expression of Zic1 mRNA was increased in WAT with cold-induced browning [84].

Zinc finger and BTB domain-containing protein 16 (Zbtb16/Zfp14) is increased in BAT during adaptive thermogenesis in mice [85,86] and also promotes the WAT browning and thermogenic function in vitro in cells.

Zinc finger protein 238 (Zfp238) expression is induced upon β-adrenergic stimulation in scWAT of mice. Zfp238 suppresses the inhibitory role of Foxo1 and increases the expression of thermogenic genes. The adipose-specific Zfp238 KO mice and 3T3-L1 cells significantly decreased Ucp1 expression [87].

PR domain zinc finger 16 (Prdm16) is a TF promoting brown and beige adipocyte differentiation and repressing the myogenic program [56,88]. The role of Prdm16 in initiating the brown/beige program versus myogenic is fulfilled by being in the same complex with histone methyltransferase Ehmt1 with its inhibitory role on the myogenic program [89]. The role of Prdm16 in inhibiting the WAT gene expression is via interacting with carboxy-terminal binding proteins, Ctbp1 and Ctbp2 co-repressor complexes [90]. Lacking Prdm16 in Myf5 positive progenitors does not affect BAT and beige development, due to the potential compensatory role of Prdm3 [91]. In the same complex with C/ebpβ and Pparγ, Prdm16 functions to promote brown/beige adipogenesis [56,92]. In addition to its role in determining brown/beige fat identity and adipogenesis, Prdm16 is also important in maintaining the brown fat identity by binding to the enhances of brown-selective genes and working together with the mediator complex to establish an enhancer-promoter loop leading to the expression of Pparα and Pgc1α [93]. Moreover, Prdm16 directly interacts with Pgc1α and induces its transcription [88,91,94]. Prdm16 also inhibits the signaling of repressor type 1 interferon response genes thereby preventing mitochondrial dysfunction and reduced Ucp1 levels [95]. Prdm16 regulates the browning of WAT as its overexpression increases beige adipocytes and thermogenesis in WAT while its deficiency inhibits beige adipocyte formation [96,97].

PR domain zinc finger 3 (Prdm3) has a complementary role to Prdm16 especially during early developmental stages in mice and, interacts with mediator complex subunit 1 (Med1) at chromatin level to regulate the brown-specific program. As a commitment factor, Prdm3 also induces the expression of Ucp1 and Pgc1α in C2C12 myogenic cells [91,93].

Pparγ co-activator 1A (Pgc1α) plays a crucial role in cold-induced thermogenesis and thermogenic maintenance in differentiated brown and beige adipocytes. Pgc1α expression is highly induced in response to the cold and upon its further activation after being phosphorylated as a downstream target of the cAMP pathway, Pgc1α interacts with several TRs including Prdm16 and Pparγ and activates the thermogenic genes [98,99]. Among others, the Pgc1α-Irf4 complex regulates the Ucp1 gene expression [100], the complex formed by thyroid hormone receptor (TR), Pgc1α, Prdm16, and Med1 also activates Ucp1 transcription [94,101]. The complex formed by Pgc1α and nuclear respiratory factors, Nrf1 and Nrf2, promotes the activation of several mitochondrial genes [102]. Pgc1α overexpression induces the thermogenesis in adipocytes and myocytes [103,104]. Brown adipocytes lacking Pgc1α express almost the same level of Ucp1 and other thermogenic genes, however, show a lower level of Ucp1 expression in response to the adrenergic stimuli [105,106]. Pgc1α is also required for the browning of WAT [107].

Interferon regulatory factor 4 (Irf4) interacts with Pgc1α upon cold stimuli and regulates the expression of Ucp1 through binding to its regulatory regions on the chromatin [100]. 

Zinc finger protein 516 (Zfp516) also increases brown adipogenesis as well as thermogenesis upon cold induction by interacting with Prdm16 which activates Ucp1 and Pgc1α gene expression [108].

cAMP-responsive element-binding and activating transcription factor 2 (Creb-Atf2); cold induction increases the adrenergic pathways as well as the intracellular levels of cAMP. This leads to PKA-dependent phosphorylation and activation of Creb and Atf2 which will further result in activation of Ucp1 and Pgc1α gene expression [109].

Forkhead box protein C2 (Foxc2) expression increases beige adipocyte formation by promoting the protein kinase A (PKA) activity that is a main downstream kinase activated by adrenergic pathway upon cold induction [110]. The Foxc2 transgenic mice that show increased mitochondrial number and respiration in scWAT do not gain weight on the high-fat diet (HFD) as compared to the control mice. Moreover, the expression of Foxc2 in 3T3-L1 cells inhibits adipogenesis by blocking the Pparγ expression [111].

Krüppel-like factor 11 (Klf11) expression is induced in vitro in human white adipocytes in response to Pparγ agonist, rosiglitazone, and via maintaining the association of Pparγ with super-enhancers of beige-selective genes, Klf11 promotes beige adipocyte-selective gene expression [112]. 

Krüppel-like factor 9 (Klf9) in vitro and in vivo in mice regulates the cold-induced browning of WAT and thermogenic function of AT through enhancing the Pgc1α expression [113].

GA-binding protein α (Gabpα) is the TF expressed in myoblasts that inhibits myogenesis and promotes adipogenesis and beige fat development. In vitro, in C2C12 myoblasts, Gabpα expression increased beige adipogenesis to the levels comparable to Prdm16. The interaction between Pgc1α and Gabpα is also shown to stimulate mitochondrial biogenesis and the OXPHOS (mitochondrial oxidative phosphorylation) program [114,115,116]. Gabpα expressing beige adipocytes unlike other beige adipocytes have a higher glucose oxidation rate than FA oxidation [70].

Nuclear receptors including the Reverbα [117,118], Errα [119], Εrrγ [119], Rxrα [120], and Nur77 [121] have been described to positively regulate the brown and beige adipose development and function.

Several TFs and activating cofactors are shown to have negative effects on beige/brown fat formation and function including Hes1 [122], Irx3 [123], Irx5 [123], Rip140 [124,125,126], Tle3 [127], Zfp423 [128,129], Hoxc8 [130], Hoxc10 [131], Twist1 [132], Foxa3 [133,134], Foxo1 [135,136], Foxp1 [137], Rb [138], Src2 (Tif2), Smad3 [139], Usf1 [140], Mrtfa [141], Lxr [142], and P107 [143,144,145]. Transcriptional repressors such as Ctbp1 and Ctbp2 [90,146] suppress the WAT gene expression and promote the browning of WAT.

### 3.2. Epigenetic Regulation of Brown and Beige Adipocytes

Epigenetic regulation is a heritable mechanism that includes DNA modifications, mainly DNA methylation, and histone modifications altering gene transcription without changes in DNA sequence. The chromatin landscape governs brown/beige differentiation and commitment, and its activation is regulated by a tight collaboration between TFs and epigenetic modifiers. 

Chromatin immunoprecipitation (ChIP) of Pparγ, the master regulator of adipogenesis, combined with deep sequencing (ChIP-seq) analysis revealed that up to 55% of Pparγ binding sites are similar among the prevalent fat types, i.e., BAT, scWAT, and vWAT with only 10% of the Pparγ binding sites being specific to BAT. Moreover, only a 10% difference in the Pparγ binding sites was recognized in BAT versus WAT upon rosiglitazone (PPARγ agonist) treatment, further confirming that beige and brown AT characteristics are acquired from small specificity of the chromatin landscape [74,112,147]. Using transgenic nuclear tagging and translating ribosome affinity purification, NuTRAP mice, and NuTRAP reporters in adipocytes, the transcriptomic and epigenomic profiles of beige, brown, and white adipocytes are defined in vivo. These strategies further confirmed the stability of chromatin landscape in BAT and the plasticity of beige adipocytes upon temperature change [148]. 

The enrichment of active histone marks such as H3K4me1/2/3 and H3K27ac at DNA regulatory regions (promoter and enhancer) promotes the expression of nearby genes. Contrarily, the recruitment of repressive histone marks, such as H3K27ac, H3K27me3, H4K20me3 to DNA regulatory regions suppresses the gene expression. Active histone marks such as H3K4me1/2 and H3K27ac are enriched in BAT and not WAT lineage enhancers [149]. Ucp1 promoters in BAT are enriched in active histone mark H3K4me3, and in WAT are enriched in H3K27me3 repressive mark [150]. The expression of repressive histone marks is diminished upon cold induction in brown adipocytes [151]. Overall, the recruitment of active histone marks to the regulatory regions of BAT-selective genes seems to play an important role in the expression of these genes. 

Several histone methyltransferases and demethylases have been identified to regulate the chromatin landscape in brown fat through alteration of the active and repressive histone marks [152,153]. For instance, the ubiquitously transcribed tetratricopeptide repeat on chromosome X (Utx) through coordinated regulation of H3K27me3 demethylation and H3K27 acetylation switches the transcriptionally repressive to the active state at the promoters of Ucp1 and Pgc1α, thereby positively modulating BAT thermogenesis [150,154]. Additionally, demethylation of H3K27me3 by Jmjd3 is also necessary for the expression of BAT-selective genes and for the development of beige adipocytes both in vitro and in vivo [150]. In response to acute cAMP stimuli, jumonji domain-containing 1A (Jmjd1a) demethylates the repressive H3K27me3 in brown adipocytes and regulates the Ucp1 gene expression [155]. Lysine-specific histone demethylase 1 (Lsd1), through interaction with Zfp516 (brown fat-enriched and cold-inducible TF), is recruited to Ucp1 and other BAT-selective genes such as Pgc1α, to work as a coactivator by demethylating H3K9 [156]. Mll4/Kmt2d co-localizes with lineage-determining TFs on active enhancers and its deletion significantly reduces the H3K4me1/2 active histone mark and polymerase II levels on enhancers which consequently impairs brown adipogenesis in mice [157]. Euchromatic histone-lysine N-methyltransferase 1 (Ehmt1) is a BAT enriched methyltransferase that controls brown adipose cell fate and its loss in brown adipocytes in vivo diminishes brown fat characteristics and induces muscle differentiation through demethylation of histone 3 lysine 9 of the muscle-selective genes [89]. Histone deacetylase 3 (Hdac3) activates estrogen-related receptor α (Errα) in BAT, which itself is governed by deacetylation of Pgc1α and is essential for the transcription of Ucp1, Pgc1α, and OXPHOS genes which are engaged and necessary for thermogenic programming [158]. Kmt5c methyltransferase regulates the expression of thermogenic genes by increasing the H4K20me3 repressive mark in the vicinity of enhanced transformation-related protein 53 (Trp53) promoters [159].

Brahma homolog related gene 1 (Brg1), a member of the SWI/SNF family plays a central role for thermogenesis on β-adrenergic activation by forming a complex with Jmjd1a and Pparγ; wherein this complex enhances Ucp1 expression by facilitating the enhancer-promoter chromatin looping [155]. Additional roles of histone modifiers, including histone acetyltransferases (Hats), histone deacetylases (Hdacs), histone methyltransferases (Hmts), and histone demethylases have been comprehensively reviewed by Nanduri [160]. 

The role of DNA (de)methylation events in beige and brown AT development and function are well discussed by others [161]. Several genes including members of the Hox family genes are identified to be differentially methylated between white and brown fat tissue implying the role of methylation in lineage specificity [162]. DNA methylation at CG sites on the Ucp1 enhancer regions is decreased with cold-induced browning in WAT. DNA methylation inhibitor, 5-azacytidine, increases the expression of Hox genes (mainly Hoxc10), thereby suppressing the browning of WAT [131,162]. In mice, Dnmt1 expression leads to the development of brown fat versus muscle by increasing the DNA methylation at the MyoD1 promoter and thereby inhibiting the expression of the muscle-specific gene, MyoD1 [163]. DNA demethylase ten-eleven translocation 1 (Tet1) inhibits the thermogenic function of BAT by suppressing the thermogenic gene, Hdac1. The expression of Tet1 is decreased with cold-induced browning of scWAT in mice, and the Tet1-KO in WAT showed enhanced thermogenic function in adipocytes as measured by the expression of the thermogenic genes including Ucp1 and Pgc1α [164].

### 3.3. Non-Coding RNAs

In addition to TFs and epigenetic regulation, non-coding RNAs including microRNAs (miRNAs) and long-noncoding RNAs (lncRNAs) have been found to play important roles in beige and brown fat commitment, differentiation, and function either by repressing or inducing the expression of genes involved in these processes. 

#### 3.3.1. MicroRNAs (miRNAs)

MicroRNAs (miRNAs) are small (21 to 25 nucleotides) non-coding RNAs, fundamental to the regulation of gene expression. They are processed by RNase III enzymes, Drosha and Dicer, in the nucleus and cytoplasm respectively. In the cytoplasm, the mature miRNA is loaded into the RNA-induced silencing complex (RISC), which contains Argonaute 2 (AGO2), the protein responsible for guiding the mature miRNA to its target mRNA. Once the miRNA-RISC complex binds to its target mRNA, translation is inhibited and, in some cases, mRNA is destabilized and degraded [165]. The importance of this pathway to adipocyte differentiation and function is evidenced by in vivo studies where deletion of AT Dicer or the Drosha’s cofactor, Dgcr8, almost completely ablated the production of mature miRNAs in the targeted cell, and altered WAT and BAT distribution, morphology and function [166]. Additionally, studies have also evaluated the contribution of individual miRNAs and described their role in beige and brown fat formation and function [167]. For instance, miR-133 represses the adipogenic lineage commitment of satellite cells by decreasing the expression of Prdm16. During cold exposure, miR-133 is downregulated leading to satellite cell-derived brown adipocytes [168]. On the other hand, miR-328 and miR-193b induce brown adipogenesis and block muscle progenitor commitment, in part, through downregulation of β-secretase Bace1 [169]. Moreover, miR-30 was shown to regulate browning of WAT, and the thermogenic function of beige and brown adipocytes by targeting Rip140, a known thermogenic corepressor [170].

#### 3.3.2. Long Non-Coding RNAs (lncRNAs)

The molecular regulation by RNAs is not limited to small RNAs and increasing evidence has suggested that long non-coding RNAs (lncRNAs) also play a role in beige and brown development and function [171]. LncRNAs are RNA molecules with more than 200 nucleotides in length and their mechanism of action includes chromatin remodeling, chromatin interactions, natural antisense transcripts (NATs], as well as interacting with RNA binding proteins in the cytosol [172]. Studies evaluating the role of lncRNAs in beige and brown fat formation and thermogenesis have observed the interaction of the lncRNA-Blnc1 with the TF of Ebf2 and the zinc finger protein Zbtb7b. Mechanistically, these factors form a ribonucleoprotein complex with lncRNA-Blnc1 and stimulate a thermogenic gene program in beige and brown adipocytes [173,174,175]. The lncRNA-BATE1 was also shown to positively regulate the beige and BAT formation. In the cytosol, lncRNA-BATE1 binds to the RNA binding protein Celf1, which is known to bind to the Pgc1α mRNA inducing its degeneration and suppressing its translation [176].

## 4. Thermogenesis Pathways and Players

The browning process involves trans-differentiation of mature white adipocytes or de novo adipogenesis of beige adipocytes. It can be induced by adrenergic stimuli, HFD feeding, and cold exposure [177,178,179,180]. This de novo adipogenesis process involves proliferation and differentiation of beige adipocytes from its progenitor pool which are present in adipose vasculature mural cells as well as smooth muscle cells that express smooth muscle actin (Sma), Myh-11, or Pdgfrα [181]. Another contribution to the overall beige fat content is by activation of dormant beige adipocytes which is also considered a trans-differentiation process as no intermediate progenitors are involved [21,67,180,182,183,184,185]. While current tracing technologies are unable of distinguishing between white to beige adipocyte trans-differentiation and the activation of dormant beige cells, Sebo and Rodeheffer have extensively discussed existing strategies available for lineage segregation of adipocytes [186].

Initiation of various signaling processes in AT may lead to thermogenic activation of BAT and browning of WAT. Several receptors on AT play a central role in such pathways leading to the increased thermogenic function of adipocytes. The physiological ligands for such receptors on adipocytes are either adipokines secreted by AT itself [187,188,189] or factors released from the various organs in response to environmental challenges such as cold, fasting, feeding, and exercise [190]. Here, we summarize the main signaling pathways described in WAT browning and thermogenic activation.

### 4.1. Adrenergic Signaling

ADRβ3 is the main receptor on AT involved in adrenergic pathways related to adaptive thermogenesis in brown or beige adipocytes. Norepinephrine (NE) released from the sympathetic nervous system (SNS) and M2 macrophages [188,191,192] is the primary ligand of ADRβ3 that upon binding activates the protein kinase A (PKA) and subsequently activates the p38 MAPK and thyroxine 5′-deiodinase leading to the induction of thermogenic gene program [137,193,194].

### 4.2. Thyroid Hormone (TH) Signaling

The main TH receptors in adipocytes are TH receptor α (TRα) that mediates synergistic effects of TH signaling and SNS, TH receptor β (TRβ) that modulates the expression of Ucp1. During the thermogenic adaptation, the thyroxine (T4) released from the hypothalamic-pituitary-thyroid axis after entering the AT will be converted into the triiodothyronine (T3) by type II thyroxine 5′-deiodinase (Dio2), an enzyme controlled by NE. Besides, TH affects the hypothalamus and promotes the AMP-kinase induction, and enhances the SNS function of NE production, leading to an increased thermogenic function [195].

### 4.3. Bile Acid Signaling

The main bile acid receptor on AT is the G-protein-coupled bile acid receptor, Gpbar1 (TGR5). It has been shown that the bile acids released from the liver after a meal and bound to the TGR5 receptors on AT also contribute to the regulation of Dio2 expression by increasing the levels of cAMP [196]. TGR5 signaling induces the WAT browning and thermogenesis by increasing lipolysis, free fatty acids (FFAs) production, and β-oxidation. TGR5 signaling also increases the number of mitochondria by inducing the mitochondrial fission (separates one into two) through the Erk/Drp1 pathway, further improving mitochondrial respiration [197].

### 4.4. Angiotensin II Signaling

The primary angiotensin II (AngII) receptors in AT are angiotensin type 1 or type 2 receptors (AT1R and AT2R). AT is a major source of AngII and, indicating the autocrine and paracrine role of AngII in regulating adipose functions and self-remodeling. The in vitro study conducted in mouse and human primary adipocytes showed that activation of AT2R increases the WAT browning and brown adipogenesis by increasing the Pparγ expression, as well as enhancing the Erk1/2, PI3kinase/Akt, and AMPK signaling pathways. In mice, AngII also contributes to the browning of WAT and enhanced thermogenesis by increasing the adiponectin release and decreasing the levels of TNFα, TGs, and FFAs in blood serum [198].

### 4.5. Fibroblast Growth Factors (Fgf) Signaling

The Fgf receptor (FgfR)/b-Klotho complexes are also located on the adipocytes cell surface in mice and humans. The main Fgfs regulating the BAT activity and WAT browning include Fgf15, Fgf19, and Fgf21 secreted from the liver, and Fgf6& Fgf9 released from the BAT. The main action of Fgf21 is conducted by inducing the expression of Pgc1α, Fgf6, and Fgf9 that involve Fgf receptor-3 (FgfR3), prostaglandin-E2, and interaction between estrogen receptor-related alpha, flightless-1 (FlII), and leucine-rich-repeat-(in FlII)-interacting-protein-1 as a regulatory complex for Ucp1 transcription. Fgf15 and Fgf19 increase the blood levels of Cxcl14 which is a batokine regulating the Ucp1 expression and thermogenesis [199,200,201].

### 4.6. BMP Signaling

BMP signaling relies on the binding of BMPs to type I and II BMP receptors on AT. The main BMPs regulating the thermogenic commitment and activity of beige and brown AT include BMP4, BMP7, and BMP-8b. Although BMPs are mainly known as adipokines, the precise source of BMP’s secretion remains elusive. BMP-8b is a batokine induced by nutritional and thermogenic factors in mature BAT which increases the thermogenic activity of BAT by; I) increasing the p38 MAPK/Creb signaling and sensitizing the BAT to NE, and II) acting on the hypothalamus increasing the AMP-activated protein kinase (AMPK) phosphorylation, leading to an anorexigenic state. BMP4 and BMP7 increase the Ucp1 expression and mitochondrial biogenesis via a p38 MAPK and Pgc1α dependent pathway [202,203,204].

### 4.7. Natriuretic Peptides (NPs) Signaling

NPs are mainly released from the heart and bind to the natriuretic peptide receptors (NPRs) on the AT. Activating the cGMP-dependent protein kinase (PKG), the action of NPs is additive to the effects of adrenergic signaling leading to BAT activation and browning of WAT [205,206,207].

### 4.8. Irisin Signaling Pathway

In rodents and humans, exercise enhances the release of the irisin from the muscle. Although the irisin receptors in fat are still debated, the irisin-induced thermogenic gene program was shown to be mediated via the integrin αV family of receptors. Irisin expression in muscle is regulated by Pgc1α expression. In AT, irisin induces the WAT browning and enhances the thermogenic activity of BAT [208,209].

## 5. Brown and Beige Adipose Tissue Function

Due to differences in the makeup and location of the fat itself, both brown and beige fat depots differ from white fat in terms of their physiological function, especially in terms of energy homeostasis and their secretory role. White fat is known to play a major role in FA biosynthesis and store energy in the form of TG whereas brown and beige fat are the important players in the process of heat dissipation/energy expenditure [210,211,212]. These contradictory roles among different types of fat can be partly attributed to the difference in the mitochondrial proteome and lipid composition discussed below. 

### 5.1. Role of Brown and Beige Fat in Thermoregulation

Unless exposed to extreme conditions or fluctuating temperatures, mammals can keep their body temperature within a narrow range which is critical for the survival of these species as the critical biochemical reactions that occur under normal physiological conditions are affected when core body temperature is disrupted [213]. Brown and beige fat are important organs involved in thermoregulation. For instance, when humans are exposed to colder temperatures, BAT mass and activity are increased resulting in increased cold tolerance [214,215,216]. On the other hand, removal of BAT and beige AT in mice using genetic approaches leads to fatal hypothermia when these mice are exposed to cold [217].

At the cellular level, brown and beige adipocyte mitochondria are rich in proteins involved in the tricarboxylic acid cycle (TCA), electron transport chain complexes I-IV, and FA oxidation [218]. The main characteristic of both brown and beige adipocytes is the presence of Ucp1 at the inner membrane of mitochondria [219]. Thermogenesis occurs when Ucp1 uncouples the proton motive force of the respiratory chain. Under normal conditions, the electron transport chain generates a proton gradient in the inner mitochondrial membrane which powers ATP production by ATPase. When Ucp1 is active, it transfers the protons generated from the electron transport chain back across this membrane, dissipating the electrochemical gradient. Oxidative metabolism that is required to maintain the normal function of the cells consumes calories and leads to an increased temperature of the cells [220]. Thermogenesis by its nature is an energy-intensive process that upon activation increases the whole-body EE. To sustain this process, activated brown and beige fat depots require a continuous supply of substrates in the form of glucose, FFAs, and intracellular TGs. This directly contributes to reduced blood glucose, improved insulin sensitivity, TG serum clearance, loss of body fat, and consequently a general improved metabolic health [221,222,223].

Although Ucp1 mediated energy dissipation and its role in metabolism and thermoregulation have been most widely discussed in the literature, it is now understood that several other thermogenic mechanisms also exist. For example, at thermoneutral temperature, deletion of Ucp1 has no effect on EE or weight gain when mice are submitted to HFD [224,225,226,227,228]. When exposed to cold, Ucp1-/- gradually adapt to the temperature and maintain their normal body temperature [108,229,230,231,232]. 

Though these Ucp1 independent mechanisms are predominantly studied in beige AT, they have also been detected in classic brown AT [233]. Some Ucp1 independent thermogenic pathways include; (I) the endogenous uncoupler peptidase M20 domain containing 1 (Pm20d1) which produces *N*−acyl amino acid and independent from Ucp1 increases mitochondrial respiration and brown and beige fat thermogenesis [234], (II) Slc25a25 which transports ATP-Mg^2+^/P[i) across the mitochondrial inner membrane and produces heat independent from Ucp1 [235], (III) the ADP/ATP carrier (AAC) in mitochondrial inner membrane mediates the proton leak from the intermembrane mitochondrial space to the matrix and dissipates the energy in the form of heat [236], (IV) the futile creatine cycle also is known to dissipate the energy and produce heat in response to cold or β-adrenergic activation in mice and humans and inactivation of this cycle reduces the thermogenic potential [237,238,239,240], (V) the futile cycling of lipolysis/re-esterification in which ATP is used to produce the triacylglycerols, diacylglycerols, or monoacylglycerols from acylglycerol is Ucp1-independent and is activated in response to adrenergic stimuli in WAT and BAT [241,242], (VI) the ATP-dependent Ca^2+^ cycling via Sarco/endoplasmic reticulum Ca^2+^-ATPase2b (Serca2b) and ryanodine receptor 2 (Ryr2) is also an Ucp1-independent thermogenic pathway. The activation of α1/β3-adrenergic receptors or the Serca2b-Ryr2 pathway enhances the Ca^2+^ cycling thereby, increases the Ucp1 independent thermogenesis [243,244], and (VII) the increase of adenine nucleotide translocase 2 (Ant2), an inner mitochondrial membrane, caused by high-fat diet feeding increases the protons leak and promotes diet-induced thermogenesis independent from Ucp1 [245,246].

### 5.2. Glucose and Lipid Metabolism by Brown and Beige Fat

In addition to endogenous TG breakdown, circulating TG-rich lipoproteins are hydrolyzed by lipoprotein lipase (Lpl) and FFAs are taken up through transporters such as Fatp1 and Cd36 to meet the high supply of fuel demand for thermogenesis [247,248]. Moreover, FFAs derived from WAT are also taken up by BAT via insulin-mediated translocation of the above-mentioned FA transporters [249]. Paradoxically, mild cold exposure induces de novo lipogenesis (DNL) and this is required for optimum BAT function. This counterintuitive mechanism is believed to be important to restore lipid droplets and may contribute to the synthesis of signaling lipids. Furthermore, enhanced DNL is known to increase the levels of cellular acetyl-CoA and malonyl-CoA, which can be a source of acetyl groups for protein lysine acetylation. This in turn may regulate epigenetic signals in BAT to sustain thermogenesis [250].

BAT and beige activation also increase insulin-independent glucose uptake, mediated by AMPK pathway and the glucose transporters Glut1 and Glut4. This leads to the hypothesis that thermogenesis activation may be used as therapy for insulin resistance and diabetes, which will be discussed later. In the cell, glucose will be utilized by the DNL pathway [251,252], stored as glycogen, or oxidized in the TCA cycle to feed the electric transport chain during thermogenesis activation [253].

### 5.3. Secretory Role of Brown and Beige Adipose Tissue

WAT is well known for its endocrine function due to the secretion of signaling molecules, so-called adipokines. These include leptin and adiponectin, and their impact on metabolism and appetite-control have been well studied. However, this secretory activity is not limited to white fat and intensive research is being carried out to gain information regarding brown and beige secretome [254,255]. Brown and beige fat are already known to release hormonal factors such as peptides (adipokines), lipids (oxylipokines), and exosomal miRNAs collectively termed as “batokines” which have autocrine, paracrine, and endocrine functions and are responsible for various processes within the human body such as EE, appetite control, lipid and glucose metabolism, insulin sensitivity, inflammation and tissue repair [256,257,258]. The secretory activity of brown and beige fat is complementary to the process of thermogenesis itself leading to further recruitment and activation of these fats. For most of the paracrine factors released from these thermogenic adipocytes, they act on cells present within the BAT such as preadipocytes, M2 macrophages, and endothelial cells consequently leading to differentiation and formation of mature adipocytes as well as increased vascularity. This intercellular communication is required for an efficient thermogenic process. There is still an ongoing debate as to how the secretory profile of beige AT differs from BAT as well as its implications. Moreover, some of the factors released by BAT have already been known to be majorly secreted by some other organs, causing an extra layer of complexity assigning an autocrine/paracrine/endocrine function to beige or brown AT.

A complete secretory profile of brown and beige fat might potentially play a role in designing therapeutic interventions for the maintenance of metabolic health. Some of these factors within the categories mentioned above and their known biological functions are disclosed in Table 1.

## 6. Brown and Beige Adipose Tissue in Obesity, Aging and Metabolic Disease

**Figure 1:**
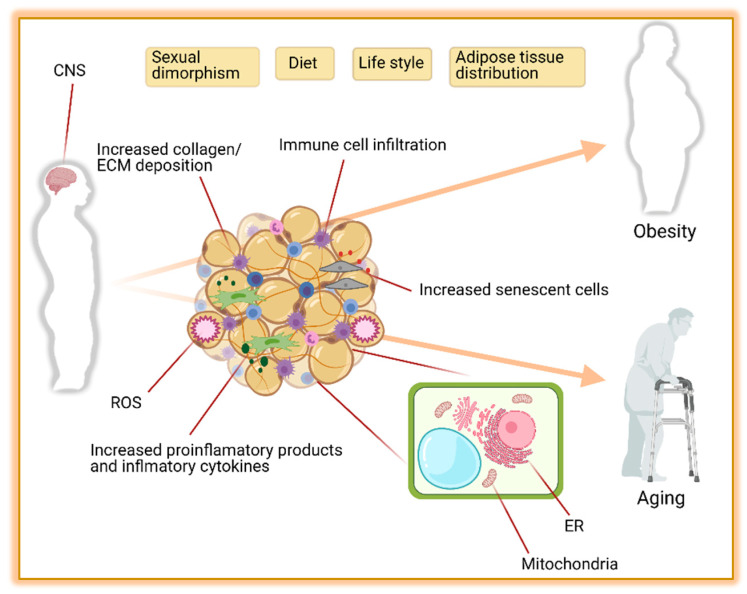
The leading causes of obesity and aging are driven by adipose tissue distribution, function, and environment. Contributions of the central nervous system (CNS), sexual dimorphism, diet, life style, and adipose tissue distribution to obesity and aging are well known. In addition, the composition of adipose tissue itself with increased collagen, extra cellular matrix (ECM), reactive oxygen species (ROS), immune cells, macrophages, and senescent cells is another major contributor to obesity and aging. Furthermore, the functionality of mitochondria and endoplasmic reticulum (ER) in adipocytes plays an important role in preventing obesity and aging complications. Figure created with ©BioRender.io.

Obesity is the major contributor to the development of metabolic diseases such as IR, T2D, dyslipidemia, and CVD. These metabolic disorders are also observed during aging [342] raising the hypothesis that unhealthy excess of body fat may accelerate the aging processes. In this regard, diet-induced obese mice are shorter-lived compared to their controls [343]. Similarly, in obese humans, the risk of premature death is increased by 1.45 to 2.76 folds [344]. The pathophysiology of obesity and aging-associated diseases are complex and share dysregulations at the cellular level [342,345]. Consistent with this, robust evidence suggests that changes in AT distribution and metabolic dysfunction are implicated in the development and disease progression during obesity and aging [346,347,348]. Here we discuss how obesity changes AT biology and its implication for the development of the metabolic syndrome. Some factors altering the AT and contributing to obesity and aging are summarized in Figure 1.

### 6.1. Adipose Tissue Distribution

In humans, AT distribution can be influenced by sexual hormones, diet, and aging. In general, females exhibit higher scWAT (gynoid fat deposition) and BAT mass, while vWAT is more preeminent in men (android fat deposition) [349]. During obesity, even though AT expansion is observed in all types of fat depots, female subjects very often present lower visceral and larger subcutaneous AT compared with males [350]. This sexual dimorphism is also observed in BAT, where BAT mass [30], and Ucp1 mRNA expression are still higher in women [351]. Genetics and hormones are the major players in sexual dimorphism [352], however, some evidence suggests that these differences persist even after menopause [30]. Interestingly, this dimorphism is associated with a lower risk to develop metabolic diseases in women and may contribute to a longer lifespan compared to men [353].

### 6.2. Metabolic Function 

It is now appreciated that AT function is also regulated in a sex-dependent manner that is widely reviewed elsewhere [349,354,355]. Here we will give an overall view of some biological processes that are impaired in the AT of obese mice and humans. These processes are interconnected and mediate the development of obesity-associated diseases.

#### 6.2.1. Sympathetic Nervous System (SNS) 

Overactivation of the sympathetic nervous system is often observed in obese subjects which contributes to the development of high blood pressure and cardiovascular diseases [356,357,358,359,360]. In AT, hyperactivation of the SNS pathway induces negative feedback, and downregulates the abundance of adrenergic receptors, decreasing the lipolytic [357], and thermogenic capacity [361]. This contributes to an increased WAT expansion, whitening of beige adipocytes [362], and decreased basal EE. Additionally, whitening of beige fat induces macrophage infiltration, brown adipocyte death and increased senescent cells, crown-like structure (CLS) formation, fibrosis, and local inflammation [362]. 

#### 6.2.2. Endoplasmic Reticulum Stress (ER) 

This organelle is composed of a membranous network responsible for the synthesis, maturation, and trafficking of proteins. It is also highly sensitive to nutrient availability. Upon nutrient overload, the increased protein synthesis followed by their misfolding and accumulation in the ER lumen induces ER stress. As a result, proteins from the unfolded protein response (UPR) Atf6, Perk, and Ire1 are recruited to reestablish the ER homeostasis [363]. In obesity, this process is hyperactivated in multiples tissues including adipose. This contributes to AT inflammation and insulin resistance [364,365]. Mechanistically, Atf6 and Perk acts through activation of NF-kB which translocate to the nucleus and induces the expression of pro-inflammatory cytokines such as IL-1 and TNFα, while Ire1a interacts with the tumor necrosis factor-a (TNFα)-receptor-associated factor 2 (Traf2), activates Jnk and IkB kinase (IKK) and downstream mediators of inflammation [363,366]. Adipocyte ER stress also leads to increased basal lipolysis through downregulation of perilipin and insulin receptor, decrease adiponectin assembling and secretion, as well as decrease in leptin release [366,367].

#### 6.2.3. Mitochondrial Dysfunction

As the central contributors to energy metabolism, mitochondria play key roles in the production of ATP, oxidative phosphorylation, production of reactive oxygen species (ROS), and Ca^2+^ homeostasis. Mitochondria also play an important role in AT homeostasis and remodeling [368,369]. The rate-limiting steps of oxidative reaction that regulate the thermogenesis in the beige adipocytes take place in mitochondria. Brown and beige fat depots are packed with mitochondria (the cells’ tiny power plants) with high expression of Ucp1 across the mitochondria inner membrane which uncouples the respiratory chain from ATP (energy) and thereby, it increases thermogenesis by heat production. The browning of the WAT is accompanied by an increase in the number of mitochondria caused by de novo biogenesis of mitochondria as well as mitochondrial fission (fission separates one into two) [370]. Contrarily, a reduced number of mitochondria resulted from mitochondrial fusion (fusion joins two mitochondria together), and mitochondrial disappearance (mitophagy) is reported during beige to white fat transition [371,372]. Mitochondrial dysfunction is present in many organs including WAT and BAT. It is characterized by increased mitochondrial DNA (mtDNA) mutations and damage, decreased oxidative phosphorylation (OXPHOS), reduced activity of metabolic enzymes, as well as changes in mitochondrial morphology, dynamics, and biogenesis [373,374,375]. In line with this, multiple symmetric lipomatosis (MSL), an adipose disorder (AD) characterized by upper body lipomatous masses, is frequently linked to multiple mutations in mitochondrial genes such as Mttk (gene encoding mitochondrial tRNA lysine involved in the assembly of proteins that carry out oxidative phosphorylation), and Mfn2 (gene encoding mitofusin 2 that helps to regulate the morphology of mitochondria by controlling the fusion process) [376,377].

#### 6.2.4. Inflammation and Endocrine Dysfunction

During obesity, adipocytes increase in size and number to accommodate the excess of nutrients in form of lipids. Excessive expansion of WAT followed by capillary rarefaction triggers a cascade of the biological processes including, ER-stress, mitochondrial dysfunction, hypoxia, changes in extracellular matrix mobility, and adipocyte death which are thought to contribute to inflammation [378]. Activation of the inflammatory response leads to the secretion of several pro-inflammatory factors TNFα, Il-1b, Il-6, and monocyte chemoattractant protein (Mcp-1) from adipocytes [379,380]. This is accompanied by infiltration of immune cells such as M1 macrophages [381], Cd8+ T cells [382], B cells [383], and eosinophils [384], thereby enhancing local and systemic inflammation [385]. The chronic low-grade inflammatory state observed in obesity is an important contributor to AT insulin resistance (IR). This is important because impaired insulin signaling in adipocytes leads to uncontrolled basal lipolysis, which can induce cell death, and also increase the circulating levels of FFAs. In turn, this leads to lipids accumulation in non-adipose organs inducing systemic IR and increasing the risk to develop cardiovascular disease and T2D [386,387,388].

## 7. Activation of Thermogenesis as Therapy for Obesity-Associated Metabolic Diseases

**Figure 2:**
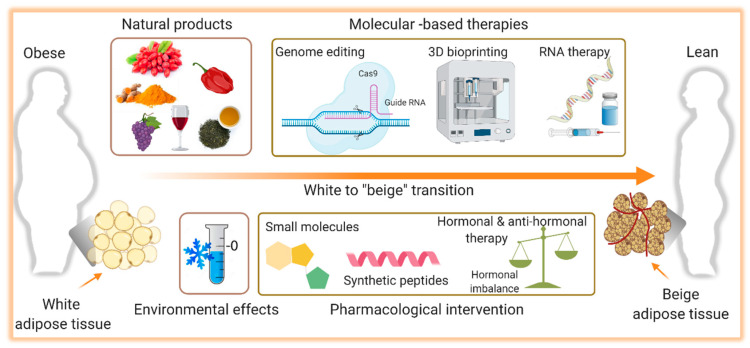
Illustration of the potential therapeutic interventions for the therapy of obesity. Induction of the browning process, the transition from white to brown-like or beige adipocytes, holds a promising therapeutic potential to combat obesity and its complications. Several pharmacological (small molecules, synthetic peptides, hormonal analogs) and non-pharmacological (natural products) interventions are known to induce browning. The role of environmental challenges such as cold exposure on white adipose tissue browning and thermogenesis is also identified. In addition, molecular-based therapies including CRIPR-based genome editing, RNA therapy, and 3D bioprinting are evolving approaches to alter the white adipocytes as a therapeutic target in obesity. Figure created with ©BioRender.io.

Over the years, the development of drugs to treat obesity was mainly focused on weight loss, primarily due to a reduction in food intake. Many of these molecules failed to meet the desired efficacy and some of them were even withdrawn from the market because of their limited success and harmful side effects [389,390]. This, with the observation that adult humans have BAT with the capability to dissipate energy, activation of BAT and thermogenesis began to be envisioned as therapy. Ever since the development of interventions that can stimulate browning of WAT as well as BAT mass increase and activation have gained greater attention and will be discussed here. A summary of the potential therapeutic interventions for obesity and metabolic disorders accompanied by aging is shown in Figure 2.

### 7.1. Cold-Induced Thermogenesis

Currently, cold exposure is the most effective intervention to activate BAT in obese humans improving whole-body insulin sensitivity and weight loss [391,392]. Some candidates have been strongly suggested to mediate the metabolic effect of BAT activation. One of the most well-investigated molecules is FGF21. This protein is mainly present in the liver, but it is also expressed in, skeletal muscle, pancreas, WAT, and BAT. Upon short-term cold exposure, FGF21 expression in adipocytes is significantly increased [393,394]. FGF21 induces browning of WAT in an autocrine manner [395] and enhances insulin signaling in the same cell [396]. Moreover, cold exposure also increases the circulating levels of FGF21 [397,398] which activates the SNS enhancing EE and weight loss [398]. 

Most recently, another member from the FGF family, FGF9, was also demonstrated to be upregulated in the scWAT and BAT of cold-exposed mice. Exerting an autocrine-paracrine regulation, FGF9 binds to FGFR3 receptor in adipocytes to regulate Ucp1 expression [399]. In addition to proteins, cold exposure induces the secretion of lipid species from BAT including 12,13-diHOME and 12-HEPE, which enhance BAT fatty acids [6] and glucose uptake [400] respectively. Altogether, cold exposure triggers an intricate metabolic network between the central nervous system (CNS) and AT which redirects the utilization of circulating glucose and FFAs to support heat production ultimately improving WAT and BAT function and whole-body metabolism.

### 7.2. Natural Thermogenic Compounds

#### 7.2.1. Berberine

Berberine is a plant-based alkaloid compound traditionally used in Chinese medicine to treat diarrhea and some infectious diseases [401]. Berberine has been extensively studied due to its potential as a cardioprotective, anti-hyperlipidemic, and antidiabetic compound [402,403]. Most recently, berberine was shown to induce Ucp1 gene expression in brown and white adipocytes through activation of 5′ AMP-activated protein kinase (AMPK) leading to an increased BAT activity, improved EE, and decreased weight gain in db/db mice [404]. More importantly, 1 month of berberine supplementation increased BAT volume and activity, reduced body weight, improved insulin sensitivity in patients with non-alcoholic fatty liver [405]. 

#### 7.2.2. Capsaicin and Capsinoids

Capsaicin and its analog capsinoids are compounds found in red peppers [406]. Several studies have shown the anti-obesity, anti-diabetic, and anti-inflammatory effects of these compounds. In rodents, capsinoids supplementation improves glucose metabolism, hepatic lipid content and enhances cold-induced EE and WAT browning [407]. In humans, chronic supplementation with capsinoids over six weeks decreased body weight and enhanced cold-induced thermogenesis in healthy adult men lacking detectable BAT, suggesting that cold exposure in combination with capsinoid ingestion recruits the activation of brown and beige adipocytes [392]. These adaptations occur through activation of the transient receptor potential cation channel subfamily V member 1 TRPV1 receptor (transient receptor potential cation channel subfamily V member 1) in the gut which sends signals to the CNS leading to β2-AR signaling activation in AT [407]. 

#### 7.2.3. Curcumin

Curcumin is a well-known flavonoid found in turmeric root. It has many therapeutic properties including, antioxidant, anti-inflammatory, anti-diabetic, and anti-obesity [408]. This is corroborated by the observation that curcumin supplementation reduces BMI, percentage of body fat, lower circulating leptin, and increased adiponectin levels in obese humans [409]. Part of these effects may be explained by the induction of browning in WAT via AMPK activation [410,411] and inhibition of preadipocyte differentiation by downregulating the Pparγ and C/ebpα [412]. In mice, supplementation with curcumin for 50 days induces higher expression of mitochondrial and thermogenic genes, higher NE levels, increased β3-AR expression in scWAT, improved cold tolerance, and lower body fat [411].

#### 7.2.4. Green Tea

Green tea is made from the leaves of Camellia sinensis and contains several different catechins, especially epigallocatechin gallate (EGCG), which accounts for about 50% to 70% of green tea catechins, and caffeine [413]. Green tea extract has several metabolic properties such as antioxidant, anti-hypertensive, anti-carcinogenic, hypocholesterolemia, and has also been shown to induce weight loss [414,415]. This evidence is supported by the reduction of body weight, mainly due to loss of vWAT mass, in obese women and men subjects submitted to catechins supplementation [414,416]. There are several potential mechanisms proposed to explain the anti-obesity effects of green tea compounds such as inhibition of de novo lipogenesis, increased FA oxidation, browning of WAT, and activation of BAT [415,417]. The effect of thermogenesis seems to be dependent on the interaction between catechins, caffeine, and NE. At the cellular level, catechins inhibit catechol-O-methyltransferase, one of several enzymes that degrade catecholamines, and caffeine inhibits phosphodiesterase resulting in higher levels of cyclic AMP (cAMP). This results in higher levels of NE and cAMP leading to fat oxidation and thermogenic activation [418].

#### 7.2.5. Resveratrol

3,5,4′-trihydroxy-trans-stilbene (Resveratrol) is a natural compound that belongs to polyphenols’ group. It is found in more than 70 different plants including grapes and has gained greater attention over the years due to its biological properties including the weight loss effect [419]. Consistent with this, resveratrol supplementation was shown to reduce the weight gain in diet-induced obese mice. This effect was mediated by improved oxidative capacity in muscle and AT and increased EE [420]. Moreover, resveratrol inhibits adipocyte differentiation and lipid accumulation [421] and induces browning of WAT [422]. The molecular effect of resveratrol is not completely understood, but some evidence suggests that interaction between AMPK activation and NAD-dependent protein deacetylase sirtuin-1 (Sirt1) leads to increased expression of Pgc1α, thereby inducing mitochondrial biogenesis [422]. In humans, the effect on weight loss and thermogenesis is not clear and differences in dose and duration of resveratrol supplementation across studies have yielded inconsistent results. Despite this limitation, some beneficial effects including improved HOMA-index have been observed 30 days after resveratrol supplementation, suggesting positive effects on insulin sensitivity [423].

### 7.3. Pharmacological Intervention 

#### 7.3.1. Beta 3-Agonist Drugs

In mice, pharmacological activation of BAT using β3-adrenoreceptor agonist drugs increases EE, reduces circulating insulin levels and body fat [424,425,426]. However, the translational potential of this approach is debatable since human β3-adrenoceptor have different binding characteristics compared to rodents and drug bioavailability also varies across species, which limits the capacity to effectively activate BAT [427,428]. Despite these limitations, a new FDA-approved drug, referred to as Mirabegron, developed to treat overreactive bladder, has been shown to improve glucose tolerance and FA oxidation. At its maximal concentration (200 mg), a single dose of Mirabegron increased BAT glucose uptake and WAT lipolysis [429]. Moreover, chronic Mirabegron treatment enhances BAT activity, induces WAT loss, increases HDL, and improves insulin sensitivity in lean and obese subjects [13,430]. Nevertheless, a recent study performed by Blondin et al. raises some concerns regarding the use of Mirabegron [431]. According to the authors, in human adipose tissue, Mirabegron seems to work mainly through b2-adrenoceptor, since b3-adrenoceptor is quite low expressed. This suggesting that this drug lacks receptor selectivity [431] and may explain some of its effects on heart rate and blood pressure [429]. 

#### 7.3.2. GLP-1 Receptor Agonist

Glucagon-like peptide 1 is a molecule secreted in response to the absorption of nutrition by the L-cells in the gastrointestinal tract. Innumerous clinical studies have demonstrated its capacity to reduce food intake, enhance insulin secretion, inhibit gluconeogenesis and improve skeletal muscle IR. Besides, recent evidence suggests that GLP-1 increased browning of WAT and BAT activation via GLP-1 binding to its receptor GLP-1R in the hypothalamus [432,433,434]. Since GLP-1 has a short half-life, GLP-1 analogs have been developed and approved as therapies to treat obesity and T2D [432]. In mice, GLP-1 analogs have the potential to induce WAT browning and BAT activation [435]. In obese and T2D humans, GLP-1 analogs enhance body weight loss and improve overall metabolism, whether this is dependent on decreased food intake or increased BAT activation yet needs to be addressed.

### 7.4. Gene Therapy 

#### 7.4.1. Ex Vivo Gene Therapy

The revolutionary approach of cellular-based therapy combined with gene editing has been considered an alternative to treat metabolic diseases and a pre-clinical study performed has shown promising results. Wang et al. used the CRISPR-Cas9 system (CRISPR-SAM) to overexpress Ucp1 in human white preadipocytes to generate the human beige/brown-like adipocytes (HUMBLE). These cells exhibit gene signatures and metabolic function similar to human brown adipocytes. Upon transplantation into mice, the HUMBLE cells differentiate into mature and functional adipocytes. Importantly, transplantation of HUMBLE cells into diet-induced obese mice resulted in increased heat production, decreased weight gain, improved insulin sensitivity, and glucose tolerance. Most strikingly, these metabolic effects were induced by the communication between the HUMBLE cells and the endogenous BAT via nitric oxide [14]. Looking forward one could envision the generation of personalized HUMBLE cells, where adipocyte progenitor cells would be isolated from the patient’s scWAT, cultivated in vitro, transformed into HUMBLE, and placed back into the patient. 

#### 7.4.2. In Vivo Gene Therapy

A more straightforward alternative to modulating the expression of a gene or a protein is the delivery of nucleotides (DNA or RNA species) to the cell of interest. Over the years, a variety of viral and non-viral methods have been developed to deliver DNA, RNA, or protein to human cells to treat different types of diseases. Currently, 12 gene therapy-based drugs are available in the market and many others are being tested in clinal trials [436], however, none of them were developed with the intent to treat obesity and its associated disease.

Hopefully, in the near future, with the use of viral vectors, we will be able to target specific tissues and overexpress a protein of interest. In line with this, one could envision the transfection of white and brown AT with the Ucp1 mRNA. A second approach will be to use the same CRISPR-Cas9 system used to generate the HUMBLE cells [14] to induce endogenous Ucp1 overexpression. The advantage of this technique compared to the others discussed earlier relies on the fact that it can be personalized, it may induce more persistent therapeutic outcomes reducing or eliminating the need for medication and avoiding any complication related to the cell transplantation.

### 7.5. 3D Bioprinting

3D bioprinting technology, allowing the construction of biological tissue in an accurate and reproducible manner is a potential approach for tissue engineering and regenerative medicine. AT bioprinting has particular needs, including morphology, composition, and heterogeneity, as well as the microenvironment, and crosstalk with other cells such as immune cells, vascularization, and ECM. 3D bioprinting of brown and beige AT aiming to create an optimal size and function and transplanting it to the patients seems like a potential strategy in the treatment of obesity and metabolic diseases. This could also be used for testing chemical and pharmaceutical products as well as evaluating the toxicity of the new drugs. Kuss et al. used 3D printed gels to test the effects of stiff vs. soft gels on immortalized human white and brown AT precursor cells and showed that white progenitors prefer soft gels to differentiate as compared to brown progenitors that their differentiation reaches an optimal level interacting with stiffer gels [437]. The feasibility of bioprinting the breast structure including the AT and mammary glands has been discussed by Chen et al., and despite several challenges including poor vascularization, it is a promising strategy to count on for the treatment of patients with breast cancer [438]. Nonetheless, most of the bioprinted tissue and organs are yet at the level of laboratory uses and there is a long way till they will be clinically applicable.

## 8. Perspectives

More than just a number on a scale or the body size, obesity is linked to many diseases and complications, including diabetes, heart disease, and many types of cancer. It is a complex dilemma and a public health concern worldwide. Activating BAT and induction of WAT browning and thereby increasing the thermogenesis is a promising strategy to improve the whole-body energy metabolism and combat obesity and its complications. In line with this, the majority of studies are performed in animals or in vitro in 2D cell cultures. Hence, the detailed mechanisms underlying the browning of WAT and BAT activation needs to be further investigated in humans. Furthermore, considering the great heterogeneity of AT, in vitro studies shall highly consider the use of 3D culture models of AT in which the native tissue function and its cellular heterogeneity would be resumed. Finally, considering the brown and beige AT as therapeutic targets, one must consider the variations that might be caused by the differences in gender, ethnicity, age, and body composition.

## Figures and Tables

**Table 1:** Secretory profile of brown and beige adipose tissue.

Type of Secretion	Physiological Function			Secreted by BAT/Beige In Vivo		Secreted by BAT/Beige In Vitro	Overall Thermogenic Effect	Target Organs	References
	Autocrine	Paracrine	Endocrine	Humans	Rodents				
**(a) Factors Released for Substrate Utilization (Lipids)**									
(1) 12,13-Dihydroxy-9Z-Octadecenoic Acid (12,13-diHOME)	YES	N/A	YES	Brown	Brown	YES	Positive	BAT, SM, H	[259,260,261,262,263]
(2) 12-hydroxyeicosa pentaenoic acid (12-HEPA)	YES	YES	YES	Brown	Brown	YES	Positive	SM, BAT	[259,264]
(3) 14-hydroxydocosahexanoic acid (14-HDHA)	YES	N/A	N/A	Brown	Brown	YES	Positive	BAT	[264]
(4) Prostaglandins (PGs)	YES	N/A	YES	Brown	Brown/ beige	N/A	Positive	WAT, BAT	[265,266,267,268]
**(b) Factors Released for Vascular Regulation**									
(1) Vascular endothelial growth factor A (VEGF-A)	YES	YES	N/A	N/A	Brown/ beige	N/A	Positive	BAT, WAT	[269,270,271,272]
(2) Nitric oxide (NO)	YES	YES	N/A	N/A	Brown/ beige	N/A	Positive	BAT, WAT	[273,274]
(3) Hydrogen peroxide (H2O2)	YES	YES	N/A	N/A	Brown/ beige	N/A	Positive	BAT, WAT	[275]
(4) Neuregulin-4	YES	YES	YES	Beige	Brown/ beige	YES	Positive	L, SNS	[276,277,278,279]
**(c) Factors Released for Regulation of Thermogenesis and Metabolic Homeostasis**									
(1) Fibroblast growth Factor 21 (FGF21)	YES	YES	YES	Brown/ beige	Brown/ beige	YES	Positive	H, P, SNS, WAT, BAT	[280,281,282,283,284,285,286,287]
(2) Fibroblast growth Factor 6& 9 (FGF6 & FGF9)	N/A	YES	N/S	Brown/ beige	Brown/ beige	Yes	Positive	BAT, WAT	[201]
(3) Endothelin-1 (ET-1)	YES	YES	N/A	N/A	Brown/ beige	YES	Negative	BAT, WAT	[288]
(4) Angiopoietin-like 8 (ANGPTL8)	YES	YES	N/A	Brown	Brown	YES	Negative	BAT	[289,290,291]
(5) Angiopoietin-like 4 (ANGPTL4)	YES	YES	N/A	Brown	Brown	YES	Negative	BAT	[292,293,294]
(6) Growth and differentiation Factor-8 (GDF-8/myostatin)	YES	YES	YES	N/A	Brown	N/A	Negative	BAT, SM	[295,296,297]
(7) Triiodothyronine (T3)	YES	N/A	?	Brown	Brown	YES	Positive	BAT	[298,299,300,301]
(8) Adenosine	YES	N/A	N/A	N/A	Brown	YES	Positive	BAT, WAT	[302]
(9) Ependymin-related protein 1 (EPDR1)	YES	YES	N/A	Brown	Brown	YES	Positive	BAT, WAT	[303]
(10) Follistatin-like 1 (FSTL-1)	YES	YES	N/A	N/A	Brown	YES	Positive	BAT	[254,304]
(11) Endocannabinoids	YES	YES	N/A	N/A	Brown	YES	Negative	BAT	[305,306,307]
(12) Low-density lipoprotein receptor relative, soluble form (sLR 11)	YES	N/A	N/A	N/A	Brown	YES	Negative	BAT	[308]
(13) SLIT2 and C-terminal fragment of SLIT2 protein (SLIT-2C)	YES	N/A	N/A	N/A	Brown/ beige	N/A	Positive	BAT, WAT	[309,310]
(14) Bone morphogenetic protein-8b (BMP-8b)	YES	YES	YES	N/A	Brown	YES	Positive	BAT, SNS	[277,311,312]
(15) Insulin-like growth factor-binding protein 2 (IGFBP-2)	YES	N/A	YES	YES	Brown/ Beige	N/A	Negative	B, BAT	[313,314,315,316,317]
(16) 3-methyl-2-oxovaleric acid	YES	YES	YES	Beige	Beige	YES	Positive	MC, BAT, WAT	[318]
(17) 5-oxoproline	YES	YES	YES	Beige	Beige	YES	Positive	MC, BAT, WAT	[318]
(18) β-hydroxyisobutyric acid	YES	YES	YES	Beige	Beige	YES	Positive	MC, BAT, WAT	[318]
**(d) Factors Released for Regulation of Immune Cells within Brown and/or Beige Adipose Tissue**									
(1) Interleukin-6 (IL-6)	YES	YES	YES	N/A	Brown/ beige	YES	Positive	MC, BAT WAT, P, H	[286,319,320,321,322]
(2) C-X-C motif chemokine ligand-14 (CXCL-14)	YES	YES	N/A	N/A	Brown	YES	Positive	BAT, MC	[323]
(3) Adiponectin	YES	YES	N/A	N/A	Beige	YES	Positive	WAT, MC	[324]
(4) Meteorin-like (METRNL)	YES	YES	N/A	N/A	Beige	YES	Positive	WAT, MC	[325]
(5) Growth and differentiation Factor-15 (GDF-15)	YES	YES	N/A	N/A	Brown/ beige	YES	Positive	BAT, MC	[326,327]
(6) Insulin-like growth Factor (IGF-1)	YES	N/A	YES	N/A	Brown	YES	Positive	BAT, L, MC	[328,329,330,331]
(7) Chemerin	YES	YES	N/A	N/A	Brown	YES	Negative	BAT, MC	[254,332,333]
**(e) Exosomal MicroRNAs**									
(1) miRNA-99b	N/A	N/A	YES	N/A	Brown	N/A	Negative	L	[334]
(2) miRNA-92a	YES	N/A	N/A	Brown	Brown	YES	Negative	BAT	[335]
**(f) Additional Regulatory Factors**									
(1) s100b and nerve growth factor (NGF)	YES	YES	N/A	Brown	Brown	YES	Positive	BAT	[336,337,338]
(2) Wingless-related MMTV integration site 10b (WNT10b)	N/A	YES	N/A	N/A	Beige	NA	Negative	BM	[313]
(3) Retinol binding protein-4 (RBP-4)	?	?	?	N/A	Brown	YES	?	-	[254,339,340,341]

SM: skeletal muscle, MC: recruitment of macrophages, WAT: white adipose tissue (induction of browning/formation of beige phenotype), SNS: sympathetic nervous system, L: liver, H: heart, BAT: brown adipose tissue, P: pancreas, B: bone (remodeling), BM: bone marrow.

## Data Availability

Not applicable.
